# Supplementary material for: Invisible Silver Nanomesh Skin Electrode via Mechanical Press Welding
Source: Nanomaterials (Basel). 2020 Mar 28;10(4):633. doi: 10.3390/nano10040633 (PMC7222014; doi:10.3390/nano10040633)
Supplement: Supplementary file 1 [file nanomaterials-10-00633-s001.pdf]

Supporting Information

Invisible Silver Nanomesh Skin Electrode via Mechanical Press Welding

Ji Soo Oh [1], Jong Sik Oh [1] and Geun Young Yeom [1,2,*]

1 School of Advanced Materials Science and Engineering, Sungkyunkwan University, Suwon 16419, Republic of Korea; jsoh9689@skku.edu (J.S.O); ojs2k@skku.edu (J.S.O)
2 SKKU Advanced Institute of Nano Technology (SAINT), Sungkyunkwan University, Suwon 16419, Republic of Korea; gyyeom@skku.edu
* Correspondence: gyyeom@skku.edu

Keywords: silver nanowire, transparent conductive film, wearable conductive film, welding, nanomesh

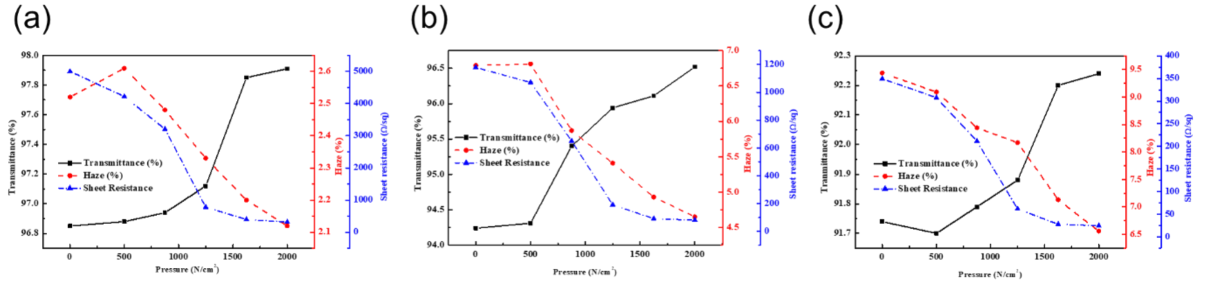

Figure S1. Change in optical transmittance (%), haziness (%), and sheet resistance (Rs, Ω/sq) for AgNW nanomesh on PMMA/SiO_2 substrate measured as a function of pressure for mechanical welding of AgNW for different spray times in of (a) 5, (b) 10, and (c) 15

For other spray times, similar mechanical welding experiments were performed for the spray times of 5, 10, and 15. As shown in Figure S1, similar changes in optical transmittance, sheet resistance, and haziness were observed.

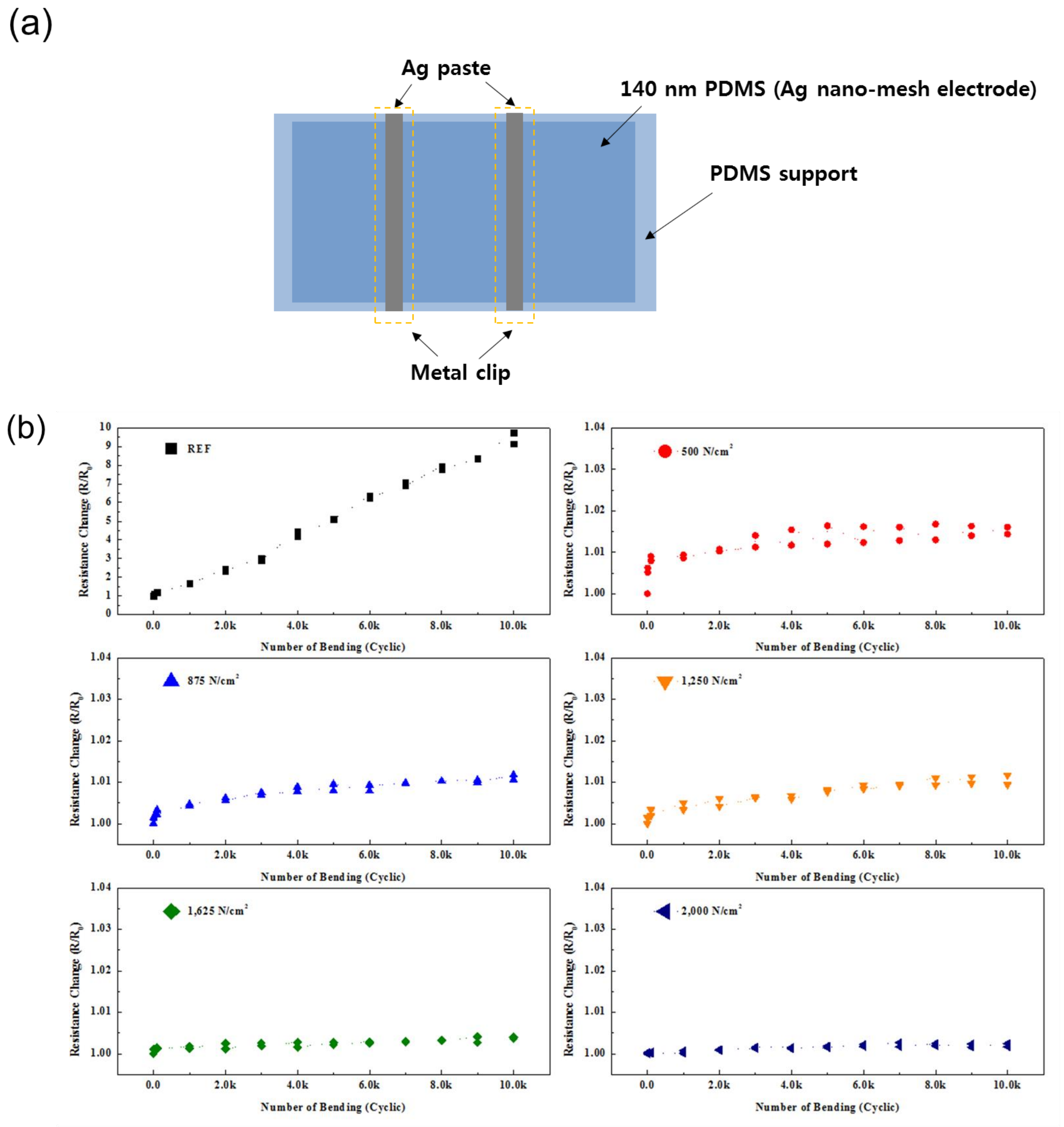

Figure S2. (a) 7 cm x 5 cm x 140 nm thick AgNW-embedded PDMS electrode (for both the 3D AgNW network and the 2D Ag nanomesh) attached to a PDMS support with two Ag paste strips for resistance measurement (b) evolution of sheet resistance change of AgNW-embedded PDMS films as a function of bending cycles (0, 500, 875, 1,250, 1,625, and 2,000 N/cm^2)

To investigate the stretch strain of the AgNW-embedded PDMS electrode, 7 cm (length) x 5 cm (width) x 140 nm (thickness) electrodes for the AgNW network and the Ag nanomesh in

Figure 4 were fabricated and, after attaching on a PDMS support and forming Ag paste stripes, the change of resistance as a function of stretch strain was measured using a four-point probe method and a lab-made bending/stretching test machine. The change of sheet resistance during the cyclic bending (radius = 3 mm) for various pressure conditions can be confirmed in more detail in Figure S2b. Each bending cycle was measured by repeating the tensile/compressive force. As shown in the graph, it can be seen that the electrodes fabricated after applying the welding process with higher pressure have higher durability than the electrodes made with the welding process with low pressure.

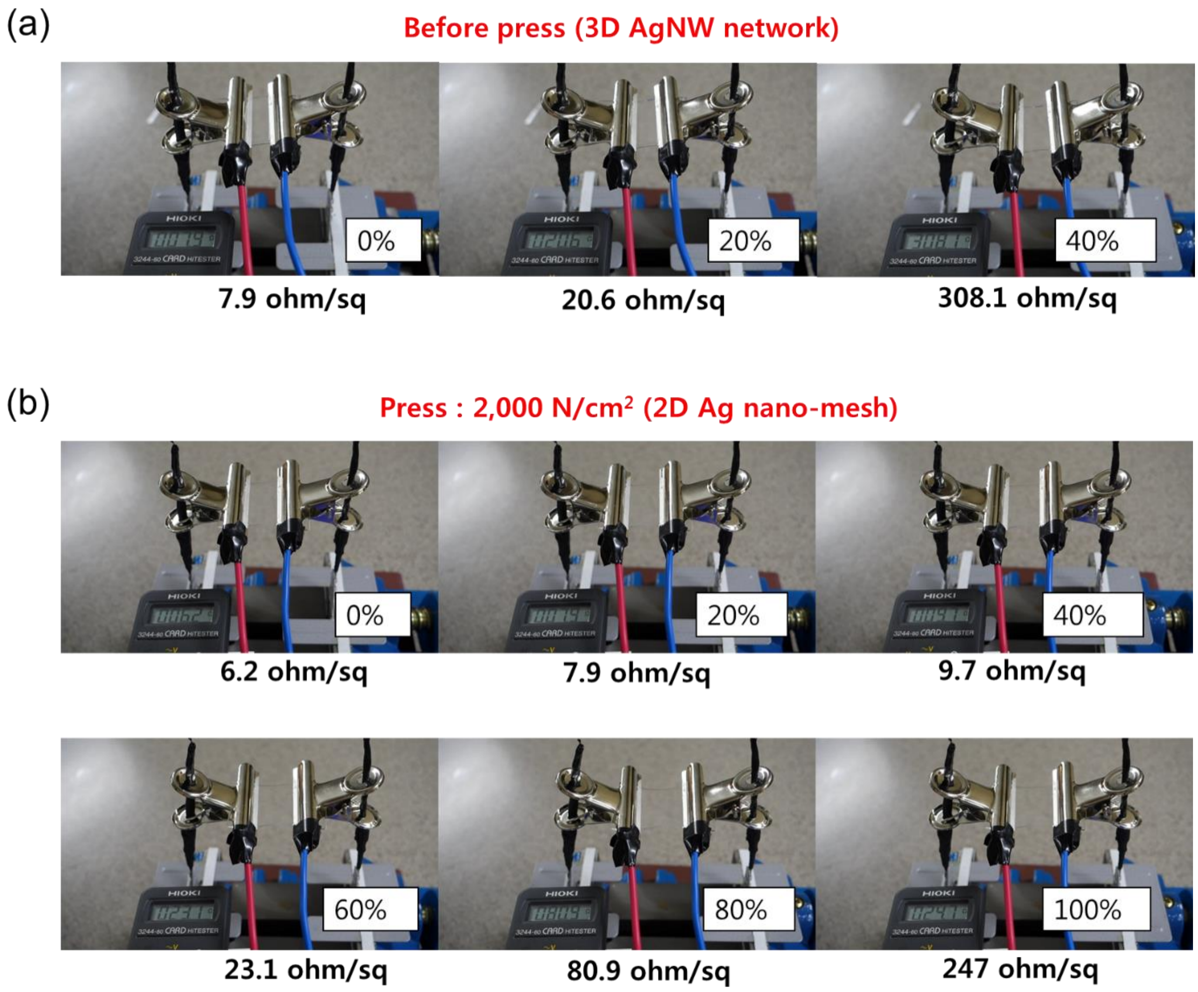

Figure S3. Photographs of the lab-made stretching test system and measured mechanical flexibility/stretchability of the AgNWs-embedded PDMS electrodes. Sheet resistance vs. strain (a) for 3D AgNW network/PDMS electrode (before pressurizing welding process) in the stretching test system and (b) for 2D Ag nanomesh-embedded PDMS electrode (after pressurizing welding process; 2,000 N/cm^2)

As shown in Figure S3, for the AgNW network-embedded PDMS, the resistance was changed from 7.9 Ω/sq at no strain to 308 Ω/sq at 40 % (~3800 % increase) while, for the Ag nanomesh-embedded PDMS, it was changed from 6.2 Ω/sq at no strain to 9.7 Ω/sq at 40 % (~56 % increase).

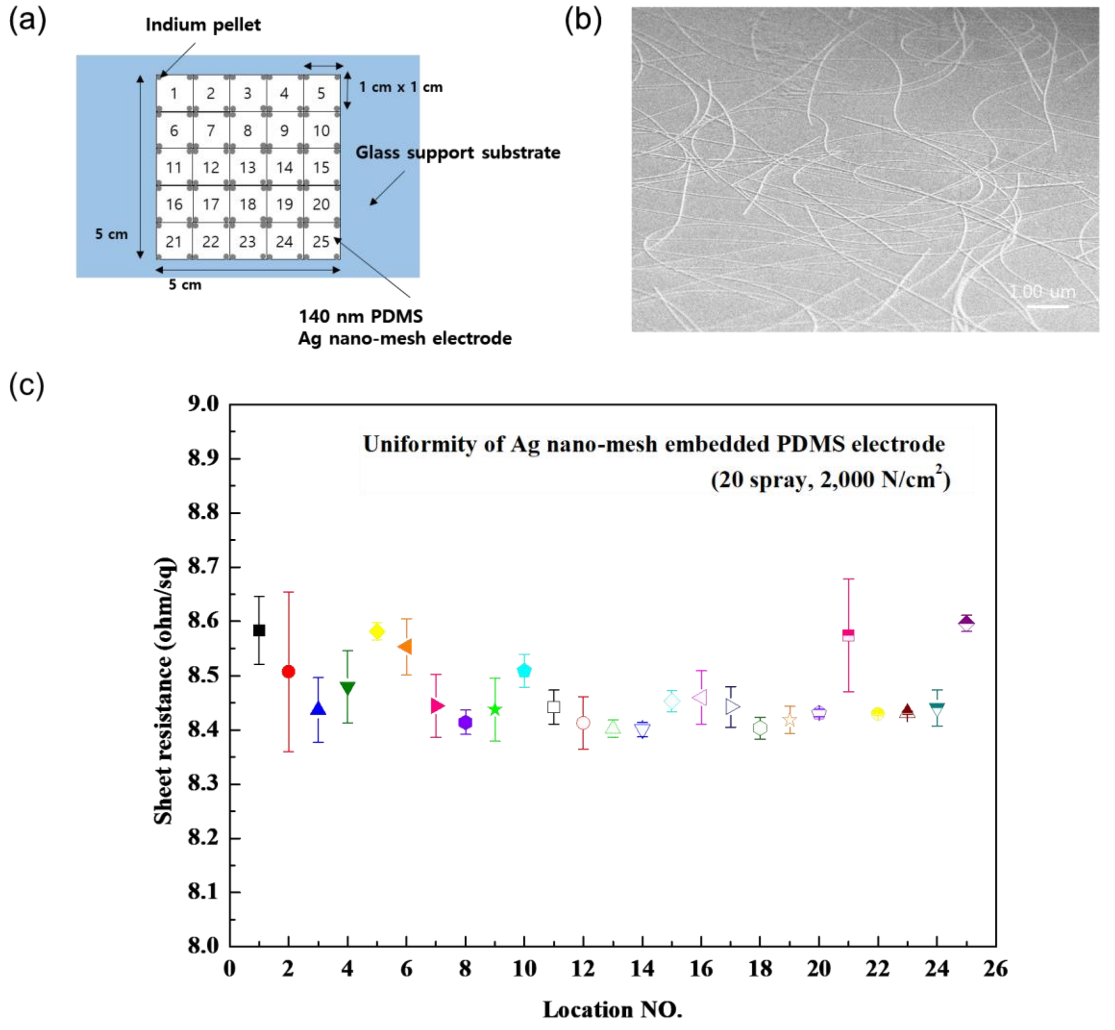

Figure S4. The electrical uniformity of the Ag nanomesh-embedded PDMS electrodes.

We fabricated an Ag nanomesh-embedded PDMS electrode with a 5 cm x 5 cm square size using the same method used in the experiment (spray times = 20, welding pressure = 2,000 N/cm^2) to check the electrical distribution uniformity. Figure S4b is a low magnification SEM image observed after pressure welding, and it shows that Ag nanomesh is evenly distributed in the whole area. After manufacturing the free standing electrode, the free standing electrode was attached to a glass support substrate and the sheet resistance was measured with a four-point probe. As shown in Figure S4c, the lowest sheet resistance (area 13) is 8.402 Ω/sq, and the

highest sheet resistance (area 25) is 8.596 Ω/sq. The overall average sheet resistance is 8.467 Ω/sq, and the total error range is 0.023, and which is considered to be a good uniformity.
